# Ixekizumab, with or without concomitant methotrexate, improves signs and symptoms of PsA: week 52 results from Spirit-P1 and Spirit-P2 studies

**DOI:** 10.1186/s13075-020-02388-5

**Published:** 2021-01-27

**Authors:** Bernard Combe, Tsen-Fang Tsai, J. Eugene Huffstutter, Aubrey Trevelin Sprabery, Chen-Yen Lin, So Young Park, Andris Kronbergs, Matthew M. Hufford, Peter Nash

**Affiliations:** 1 Department of Rheumatology, CHU Montpellier, Montpellier University, 34090 Montpellier, France; 2 National Taiwan University, Taipei, Taiwan; 3 Arthritis Associates, Hixson, TN USA; 4 Eli Lilly and Company, Indianapolis, IN USA; 5 School of Medicine, Griffith University, Brisbane, Australia

**Keywords:** Psoriatic arthritis, Ixekizumab, cDMARDs, Methotrexate

## Abstract

**Background:**

The efficacy and safety of ixekizumab (IXE) with and without continuous concomitant methotrexate (MTX), for up to 52 weeks of treatment, were evaluated in patients with active psoriatic arthritis (PsA).

**Methods:**

Patients with active PsA who were biologic-naive (SPIRIT-P1) or had prior inadequate response to tumor necrosis factor inhibitors (SPIRIT-P2) were randomized to 80 mg IXE every 4 (IXE Q4W) or 2 weeks (IXE Q2W), after a 160-mg initial dose. In this post hoc analysis, efficacy and safety were assessed up to week 52 in the subgroups of patients who received (i) IXE as monotherapy and (ii) IXE along with a stable dose of MTX (no dose tapering or increase). Efficacy outcomes included, but were not limited to, the percentage of patients achieving the American College of Rheumatology (ACR) responses.

**Results:**

Out of 455 patients initially randomized to IXE, 177 (38.9%) received monotherapy, 230 (50.5%) had concomitant MTX use, and 48 (10.5%) had other concomitant medication. Overall, 183 (40.2%) received IXE with a stable dose of concomitant MTX for 1 year. At week 52, the percentage of patients achieving ACR20/50/70 responses in IXE Q4W monotherapy versus concomitant MTX groups were 66.3% versus 55.3%, 48.4% versus 38.8%, and 35.8% versus 27.1%, respectively; these responses were generally similar with IXE Q2W. The safety profiles were similar between patients receiving IXE with or without concomitant MTX.

**Conclusions:**

In this post hoc analysis, treatment with IXE demonstrated sustained efficacy in patients with PsA up to 1 year of treatment, with or without concomitant MTX therapy.

**Trial registration:**

ClinicalTrials.gov NCT01695239 and NCT02349295.

**Supplementary Information:**

The online version contains supplementary material available at 10.1186/s13075-020-02388-5.

## Background

Psoriatic arthritis (PsA) is a chronic inflammatory condition that predominantly affects peripheral joints and is associated with peri-articular and extra-articular manifestations. Treatment of this disease can be challenging because of its known complex nature and heterogeneous presentation [1, 2].

The current treatment guidelines from the Group for Research and Assessment of Psoriasis and Psoriatic Arthritis suggests tumor necrosis factor inhibitors (TNFi) along with disease-modifying antirheumatic drugs (DMARDs, such as methotrexate [MTX]) as first-line treatment for PsA, whereas the European League Against Rheumatism (EULAR) 2019 recommends using conventional synthetic DMARDs followed by TNFi for the treatment of PsA [3, 4]. Methotrexate is approved for the treatment of psoriasis [5]. It is used as a first-line treatment for rheumatoid arthritis and is also widely used off-label for the treatment of PsA either as monotherapy or in combination with biologics such as TNFi. However, there are limited data to establish its efficacy in the treatment of PsA by itself [6]. In the methotrexate in psoriatic arthritis (MIPA) trial, no statistically significant difference was found between MTX-treated patients and placebo-treated patients [7].

Several studies have evaluated the clinical benefit of MTX with TNFi, but the efficacy of this combination therapy remains unclear [8–13]. The Norwegian DMARD (NOR-DMARD) trial found no additional benefit of adding MTX to TNFi [10]. The Danish Biologics (DANBIO) registry found that the American College of Rheumatology 20% response rate (ACR20) was numerically higher in patients treated with TNFi and MTX compared to TNFi alone [14]. The recent SEAM-PsA trial evaluated etanercept monotherapy and combination therapy with etanercept and MTX. Overall, the findings of the trial indicated that the combination therapy with etanercept and MTX did not improve the efficacy of etanercept as measured by ACR responses [15].

Ixekizumab (IXE) is a high-affinity monoclonal antibody that selectively targets interleukin (IL)-17A [16]. The United States Food and Drug Administration and the European Medical Agency have approved IXE for the treatment of PsA with the recommended dose of 160 mg by subcutaneous injection (two 80-mg injections) at week 0, followed by 80 mg every 4 weeks (IXE Q4W) thereafter [17, 18]. Ixekizumab has been demonstrated to improve the signs and symptoms of PsA in patients who were biologic-naive (SPIRIT-P1) or had previous inadequate response or intolerance with TNFi (SPIRIT-P2) [19, 20]. The efficacy and safety data of IXE with and without MTX up to week 24 from SPIRIT-P1 and SPIRIT-P2 trials have been previously reported [21, 22]. The findings from these studies showed that IXE improved measures of disease activity and physical function when used with or without concomitant MTX therapy relative to placebo. This paper details the extent of concomitant MTX treatment modification as well as the efficacy and safety for patients treated with IXE alone and IXE with MTX for up to 1 year of treatment.

## Methods

### Study design

This post hoc analysis includes integrated data derived from 2 randomized, double-blind, placebo-controlled, phase 3 trials in patients with active PsA: SPIRIT-P1 [NCT01695239] [19] and SPIRIT-P2 [NCT02349295] [20]. The detailed study designs of these trials have been published previously [19, 20]. Briefly, patients were randomized to placebo (data not reported here), adalimumab 40 mg (active reference arm up to week 24 in SPIRIT-P1 only), IXE 80 mg every 2 weeks (IXE Q2W), or IXE Q4W. Both IXE regimens received a 160-mg starting dose. At week 16, inadequate responders (defined as < 20% improvement from baseline in both tender joint counts [TJC] and swollen joint counts [SJC]) were required to add or modify concomitant medications and were considered non-responders for the remainder of the double-blind treatment period (i.e., up to week 24). Patients were discontinued from the study if they did not meet the predefined response criteria (i.e., failure to demonstrate at least a 20% improvement from baseline in both TJC and SJC) at week 32 or any subsequent visit during the study.

Patients receiving MTX were required to (1) have been treated for at least 12 weeks prior to baseline and should be on a stable dose for at least 8 weeks prior to baseline, (2) have received oral or parenteral MTX up to 25 mg/week, and (3) continue the medication without any modification to the treatment regimen during the double-blind treatment period (weeks 0–24). Additionally, patients could have received a prior treatment with 1 or more subsequent conventional DMARDs (cDMARDs, such as MTX), and they could also undergo modification of the concomitant medication after week 24 or use other cDMARDs. A combination of cDMARDs was not allowed. In the current report, we present results for only the subgroup of those patients who were on a stable dose of MTX up to week 52. The ACR responses of patients randomized to IXE who had concomitant MTX at baseline (regardless of MTX dose modification afterwards) are included in Additional file 1.

SPIRIT-P1 and SPIRIT-P2 were conducted in accordance with Good Clinical Practice, the principles of the Declaration of Helsinki, and local laws and regulations. SPIRIT-P1 was approved by the Western Institutional Review Board (approval #1-838258-1), and SPIRIT-P2 was approved by the Bellberry Human Research Ethics Committee (Application #2015-01-049-AA). For both studies, approval was also obtained from each additional site. All patients in both studies gave written informed consent. The full lists of investigators and sites are provided in the primary manuscript supplements [19, 20].

### Study population

Adult patients from SPIRIT-P1 and SPIRIT-P2 trials with active PsA (defined as the presence of ≥ 3 of 68 tender joints and ≥ 3 of 66 swollen joints) who met the Classification Criteria for PsA were included in this analysis. In SPIRIT-P1, patients were biologic DMARD-naive. In SPIRIT-P2, patients had to have an inadequate response to at least 1 cDMARD and were required to have an inadequate response or intolerance to 1 or 2 TNFi.

In this report, efficacy was assessed up to week 52 for the subgroups of patients who received (i) IXE as monotherapy (without concomitant cDMARDs) and (ii) a stable dose of MTX from weeks 0 to 52. Additionally, ACR responses for patients who were randomized to IXE and had concomitant MTX use at baseline were assessed up to week 52.

### Assessments

Efficacy outcome measures included the percentage of patients achieving ACR20/50/70 responses, minimal disease activity (MDA), and disease activity in psoriatic arthritis (DAPSA) low disease activity (LDA) (score ≤ 14).

The ACR20/50/70 responses were defined as ≥ 20%/≥ 50%/≥ 70% improvement from baseline in the number of tender joints (TJC and SJC) as well as ≥ 20%/≥ 50%/≥ 70% improvement in at least 3 of the 5 ACR components as described previously [23]. Minimal disease activity was defined as patients achieving at least 5 of the 7 following criteria: TJC ≤ 1, SJC ≤ 1, Psoriasis Area and Severity Index Improvement (PASI) ≤ 1, or body surface area involvement ≤ 3%; Patient’s Assessment of Pain visual analog scale (VAS) ≤ 15; Patient’s Global Assessment of Disease Activity (PatGA) VAS ≤ 20; Health Assessment Questionnaire-Disability Index score ≤ 0.5; and tender entheseal points ≤ 1 [24, 25]. The DAPSA LDA score of ≤ 14 was measured by the sum of TJC, SJC, high sensitivity C-reactive protein, PatGA VAS (0–10 cm scale), and Patient’s Assessment of Pain VAS (0–10 cm scale) [26, 27].

Safety outcomes included the proportion of patients experiencing treatment-emergent adverse events (TEAEs), serious adverse events (SAEs), adverse events (AEs) leading to discontinuation, and prespecified AEs of special interest.

### Statistical methods

This post hoc, integrated subgroup analysis included all patients initially randomized to IXE at week 0 from the intent-to-treat populations of the SPIRIT-P1 and SPIRIT-P2 trials. All patients who discontinued from treatment before week 52 were also included in the analysis.

This analysis was conducted for the IXE Q2W and IXE Q4W groups separately. Patients receiving concomitant cDMARDs other than MTX at the time of randomization and those who had any MTX dose change at any point during weeks 0 to 52 were excluded from the efficacy and safety analysis. Missing values were imputed using non-responder imputation for categorical analyses. As an additional analysis, ACR20/50/70 responses were analyzed among patients randomized to IXE at baseline and who took concomitant MTX at study initiation irrespective of any dose change at the post-baseline period. This analysis included a broader patient population who changed MTX dose due to inadequate response criterion at week 16 or due to investigators’ decision between week 24 and 52 (Figure S1 (Additional file 1)).

Safety analyses were conducted on the safety population, defined as all patients who received at least 1 dose of IXE, and grouped by the defined analysis subgroups.

## Results

A total of 455 patients were included in this analysis, of whom 177 (38.9%) patients received IXE monotherapy (i.e., with no concomitant cDMARDs) for up to a year of treatment. Of the 230 (50.5%) patients who received MTX at some point through week 52, 183 (40.2%) patients received IXE with a stable dose of concomitant MTX up to week 52. Forty-eight (10.5%) patients received cDMARDs other than MTX and were not included in this analysis.

The number of patients (*n* = 47) undergoing MTX dose tapering/modification up to week 52 included the following: 5 (1.1%) increased MTX dosing, 8 (1.8%) added MTX to their IXE therapy, 9 (2.0%) discontinued MTX therapy and restarted it later (at a higher or lower dose), 14 (3.1%) tapered their MTX dosing, 9 (2.0%) discontinued MTX therapy by week 52, and 2 (< 1%) had missing data. Generally, a similar proportion of patients modified the MTX use between the treatment regimens; however, more patients in the IXE Q2W group tapered while more patients in the IXE Q4W group discontinued MTX.

Patient demographics and baseline characteristics were similar across IXE Q4W or Q2W monotherapy versus concomitant MTX groups (Table 1). The stable average dosing of MTX for the IXE Q4W group was 15.7 mg/week and for the IXE Q2W group was 16.0 mg/week (Table 2).

**Table 1:** Baseline demographics and disease characteristics

	SPIRIT-P1 and SPIRIT-P2			
	IXE Q4W		IXE Q2W	
	No MTX/cDMARDs (***N*** = 95)	MTX^**a**^ (***N*** = 85)	No MTX/cDMARDs (***N*** = 82)	MTX^**a**^ (***N*** = 98)
Age, years	51.2 (12.3)	52.0 (12.4)	52.0 (12.0)	49.1 (11.7)
Male, *n* (%)	46 (48.4)	41 (48.2)	38 (46.3)	44 (44.9)
Weight, kg	87.0 (22.6)	87.2 (18.1)	85.0 (21.9)	83.3 (18.1)
Time since PsA diagnosis, years	10.5 (9.6)	6.5 (6.6)	9.2 (9.0)	8.6 (7.0)
Patients with specific disease characteristics, *n* (%)				
Enthesitis^b^	53 (55.8)	50 (58.8)	53 (65.4)^^^	58 (59.8)^f^
Dactylitis^c^	26 (27.4)	22 (25.9)	16 (19.5)	23 (23.7)^f^
Current psoriasis^d^	93 (97.9)	80 (94.1)	77 (93.9)	91 (92.9)^^^^
Baseline disease and quality of life scores				
TJC (68 joints)	21.8 (13.6)	20.6 (15.1)	25.2 (16.6)	21.7 (15.1)
SJC (66 joints)	12.4 (8.9)	11.7 (10.8)	13.7 (10.0)	11.9 (8.1)
PGA VAS	60.1 (19.3)	57.9 (20.6)	62.9 (19.1)	61.9 (16.4)
PatGA VAS	66.5 (18.8)	64.0 (22.0)	64.9 (21.4)	63.8 (20.6)
Patients assessment of pain VAS	63.0 (20.1)	63.0 (21.6)	61.5 (21.5)	62.1 (21.7)
HAQ-DI	1.2 (0.6)	1.2 (0.6)	1.2 (0.6)	1.2 (0.6)
hsCRP, mg/L	16.6 (27.0)	15.2 (21.6)	15.6 (29.3)	14.1 (22.8)
PASI	7.5 (8.6)	6.3 (6.4)	6.9 (7.9)	6.0 (8.6)
% BSA^e^	13.9 (18.8)	14.9 (16.0)	10.6 (14.7)	13.1 (19.6)

Data are mean (standard deviation) unless stated otherwise

^a^Patients with stable dose of MTX from weeks 0 to 52 only

^b^Baseline enthesitis defined as a baseline LEI score > 0

^c^Baseline dactylitis defined as a baseline LDI-B score > 0

^d^Current psoriasis as assessed by physician

^e^Patients with psoriasis at baseline

^f^The number of patients evaluated was *N* = 97

^^^Number of patients evaluated in this group was 81

^^^^Number of patients evaluated in this group was 98

*Abbreviations*: *BSA* body surface area involvement, *cDMARD* conventional disease-modifying antirheumatic drug, *HAQ-DI* Health Assessment Questionnaire-Disability Index, *hsCRP* high sensitivity C-reactive protein, *IXE Q2W* 80 mg ixekizumab every 2 weeks, *IXE Q4W* 80 mg ixekizumab every 4 weeks, *LDI-B* Leeds Dactylitis Index-Basic, *LEI* Leeds Enthesitis Index, *MTX* methotrexate, *N* number of patients in each group, *n* number of patients in specific group, *PASI* Psoriasis Area and Severity Index, *PatGA* Patient’s Global Assessment of Disease Activity, *PGA* Physician’s Global Assessment of Disease Activity, *PsA* psoriatic arthritis, *SJC* swollen joint count, *TJC* tender joint count, *VAS* visual analog scale

**Table 2:** Summary of MTX use between weeks 0 and 52 in patients with stable MTX dose

	SPIRIT-P1 and SPIRIT-P2	
	IXE Q4W (***N*** = 229)	IXE Q2W (***N*** = 226)
Patients with stable MTX dose, *n* (%)	85 (37.1)	98 (43.4)
Overall average dose, mg/week	15.7	16.0
Route of MTX administration:		
Intramuscular, *n* (%)	1 (1.2)	2 (2.0)
Average dose, mg/week	25.0	12.5
Subcutaneous, *n* (%)	14 (16.5)	15 (15.3)
Average dose, mg/week	17.7	19.7
Oral, *n* (%)	70 (82.4)	81 (82.7)
Average dose, mg/week	15.2	15.4

*Abbreviations*: *IXE Q2W* 80 mg ixekizumab every 2 weeks, *IXE Q4W* 80 mg ixekizumab every 4 weeks, *MTX* methotrexate, *N* number of patients in each group, *n* number of patients in specific group

**Fig. 1:**
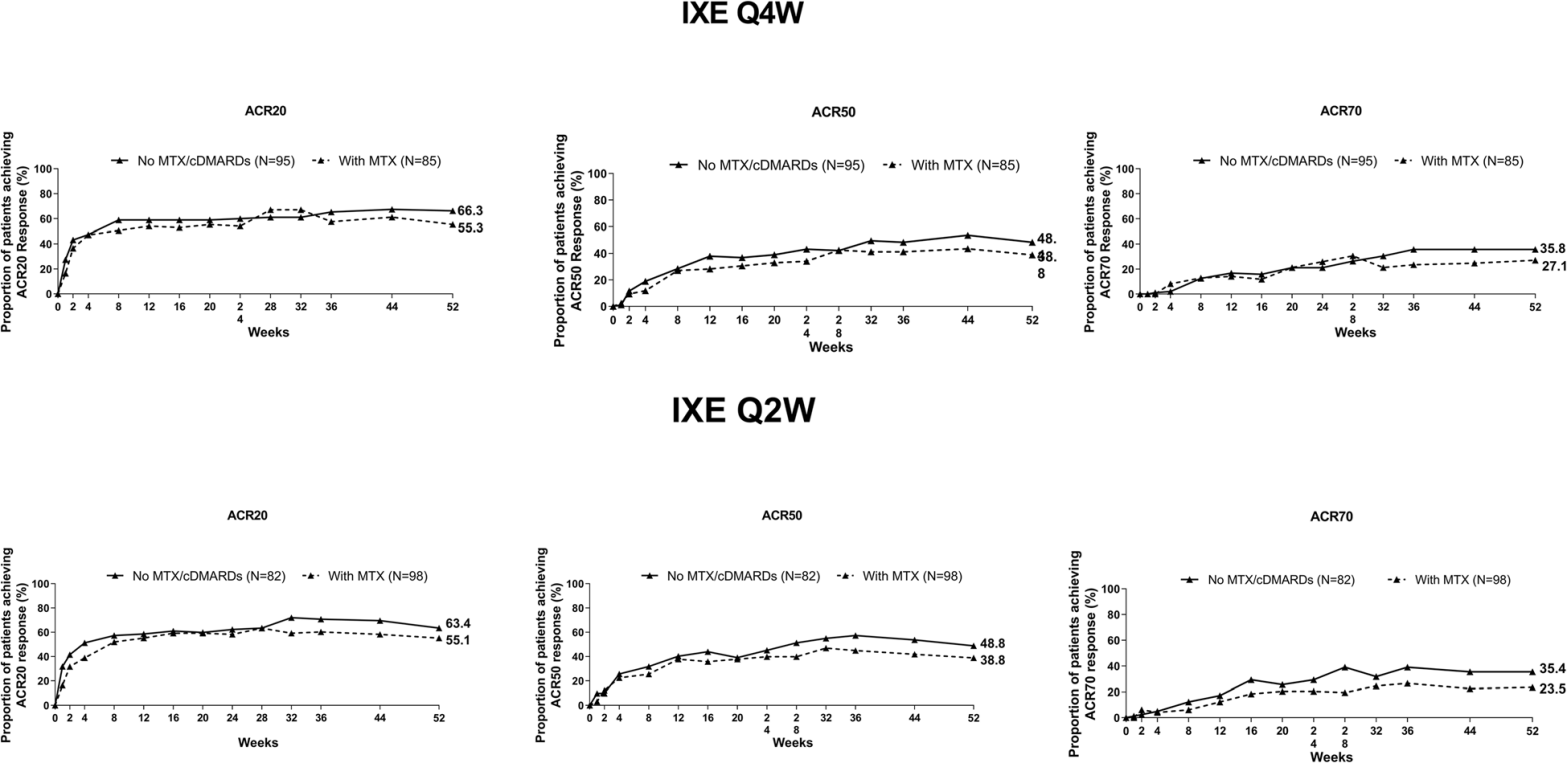
ACR20/50/70 responses with IXE with or without concomitant MTX after 52 weeks of treatment. Abbreviations: ACR20/50/70 American College of Rheumatology criteria 20%/50%/70% improvement, cDMARD conventional disease-modifying antirheumatic drug, IXE ixekizumab, IXE Q2W 80 mg ixekizumab every 2 weeks, IXE Q4W 80 mg ixekizumab every 4 weeks, and MTX methotrexate

Generally, ACR20/50/70 responses were similar or higher in patients receiving IXE Q4W or Q2W monotherapy compared with those receiving stable dose concomitant MTX during the double-blind treatment period (up to week 24). Although not formally tested for comparison from week 36 onward, the proportion of patients achieving ACR responses was higher in patients receiving IXE Q4W or Q2W monotherapy compared with those receiving stable dose concomitant MTX (Fig. 1). At week 52, the ACR20/50/70 response rates in IXE Q4W monotherapy and stable dose concomitant MTX groups were 66.3% and 55.3%, 48.4% and 38.8%, and 35.8% and 27.1%, respectively. Similar responses were seen for IXE Q2W monotherapy compared with stable dose concomitant MTX groups (Fig. 1). In patients randomized to IXE and receiving MTX at baseline irrespective of subsequent dose change, there was no apparent increase in the percentage of patients achieving ACR20/50/70 responses at week 52 relative to patients receiving IXE as monotherapy. At week 52, in IXE Q4W and Q2W treatment arms in patients with concomitant MTX use at baseline, the response rates for ACR20/50/70 were 56.1% (both groups), 40.2% and 38.6%, and 26.2% and 23.7%, respectively (Figure S2 (Additional file 1)).

Week 52 change from baseline in TJC and SJC for patients with IXE monotherapy and those receiving stable dose concomitant MTX are presented in Table S1.

**Fig. 2:**
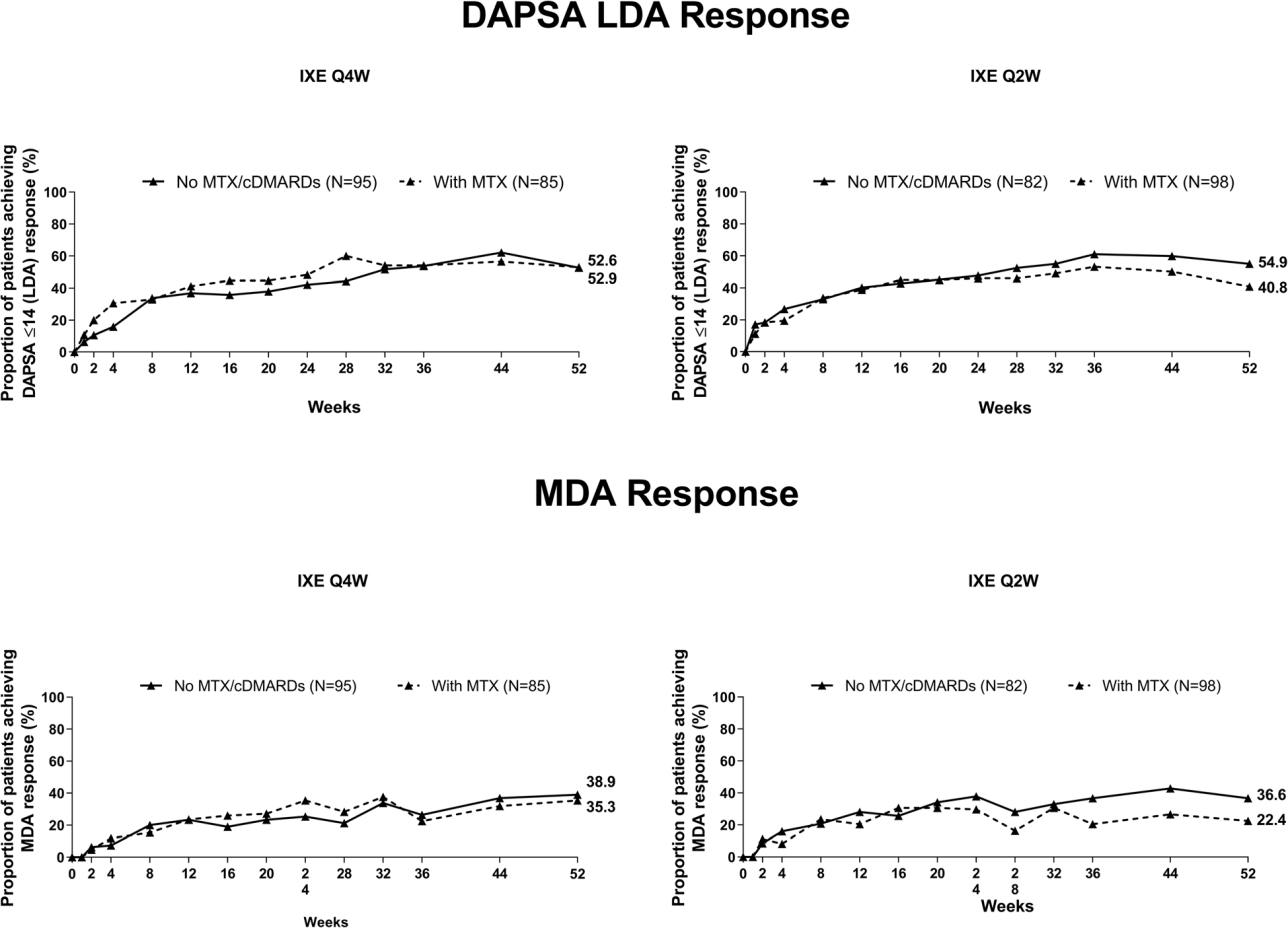
DAPSA LDA^a^ and MDA^b^ responses with IXE with or without concomitant MTX after 52 weeks of treatment. ^a^DAPSA LDA requires DAPSA score ≤ 14. ^b^MDA criteria requires improvement in ≥ 5 of 7 domains (TJC ≤ 1, SJC ≤ 1, PASI ≤ 1 or percentage of BSA affected ≤ 3, patient’s assessment of pain VAS ≤ 15, patient’s global assessment of disease activity VAS ≤ 20, HAQ-DI ≤ 0.5, and tender entheseal points ≤ 1

At week 52, the DAPSA LDA response rates in IXE Q4W monotherapy versus stable dose concomitant MTX groups were 52.6% versus 52.9%, respectively, whereas the DAPSA LDA response rates in IXE Q2W monotherapy versus stable dose concomitant MTX groups were 54.9% versus 40.8%, respectively. Overall, the proportion of patients achieving disease control (as measured by DAPSA LDA or MDA was similar in patients receiving IXE Q4W/Q2W monotherapy relative to patients receiving concomitant stable dose MTX therapy up to week 52 (Fig. 2). Although not formally tested for comparison, the proportion of patients achieving MDA or DAPSA LDA was higher in those receiving IXE Q2W monotherapy compared with those receiving IXE Q2W and stable dose concomitant MTX.

Throughout 52 weeks of treatment, the proportion of patients reporting TEAEs was similar between IXE Q4W groups with or without MTX. A higher proportion of patients who received IXE Q2W alone experienced TEAEs compared to those receiving concomitant MTX; however, TEAEs in general were rated mild or moderate in severity. Adverse events leading to discontinuation and SAEs were generally similar in either dosing regimen with or without MTX. Adverse events of special interest including injection site reactions, infection, and hepatic event were generally similar in both dosing regimens of IXE with or without MTX (Table 3). Treatment-emergent AEs of diarrhea, nausea, and headache were reported similarly between patients receiving IXE dosing with or without MTX. Treatment-emergent abnormalities in laboratory values of whole blood neutrophils, platelets, and leukocytes as well as aspartate and alanine aminotransferase levels were similar or had no elevation between patients receiving IXE dosing with or without MTX (data not shown).

**Table 3:** Safety overview of IXE with or without concomitant MTX after 52 weeks of treatment

	SPIRIT-P1 and SPIRIT-P2			
	IXE Q4W (***N*** = 229)		IXE Q2W (***N*** = 226)	
	No MTX/cDMARDs (***N*** = 95)	MTX^**a**^ (***N*** = 85)	No MTX/cDMARDs (***N*** = 82)	MTX^**a**^ (***N*** = 97)
TEAEs (≥ 1)	75 (78.9%)	67 (78.8%)	71 (86.6%)	77 (79.4%)
Mild	31 (32.6%)	39 (45.9%)	32 (39.0%)	35 (36.1%)
Moderate	39 (41.1%)	24 (28.2%)	32 (39.0%)	35 (36.1%)
Severe	5 (5.3%)	4 (4.7%)	7 (8.5%)	7 (7.2%)
SAEs	6 (6.3%)	5 (5.9%)	4 (4.9%)	3 (3.1%)
Discontinuations due to AE	5 (5.3%)	2 (2.4%)	6 (7.3%)	9 (9.3%)
AEs of special interest				
Cytopenias	1 (1.1%)	3 (3.5%)	3 (3.7%)	1 (1.0%)
Hepatic events	6 (6.3%)	2 (2.4%)	3 (3.7%)	9 (9.3%)
Infections	50 (52.6%)	37 (43.5%)	41 (50.0%)	47 (48.5%)
Injection-site reactions	20 (21.1%)	14 (16.5%)	23 (28.0%)	26 (26.8%)
Allergic reactions/hypersensitivities	8 (8.4%)	4 (4.7%)	8 (9.8%)	7 (7.2%)
Non-anaphylaxis	8 (8.4%)	4 (4.7%)	8 (9.8%)	7 (7.2%)
Malignancies	2 (2.1%)	0	0	0
Depression	2 (2.1%)	4 (4.7%)	2 (2.4%)	2 (2.1%)

Data presented are *n* (%)

Note: There were no cases of anaphylaxis, cerebro-cardiovascular events, MACE, ILD, IBD, CD, and UC observed in these subpopulations

*Abbreviations*: *AEs* adverse events, *CD* Crohn’s disease, *cDMARD* conventional disease-modifying antirheumatic drug, *IBD* inflammatory bowel disease, *ILD* interstitial lung disease, *IXE Q2W* 80 mg ixekizumab every 2 weeks, *IXE Q4W* 80 mg ixekizumab every 4 weeks, *MACE* major adverse cerebro-cardiovascular events, *MTX* methotrexate, *N* number of patients in each group, *n* number of patients, *SAEs* serious adverse events, *TEAEs* treatment-emergent adverse events, *UC* ulcerative colitis

^a^Patients with stable dose of MTX from weeks 0 to 52 only

## Discussion

This analysis concluded that IXE is efficacious in improving the signs and symptoms of PsA up to 52 weeks of treatment, whether used alone or in combination with MTX. Until week 36, ACR20/50/70 responses were either similar or higher between IXE monotherapy and concomitant MTX groups except at certain time points. From weeks 36 to 52, a higher number of patients in both IXE monotherapy groups achieved ACR20/50/70 responses compared to those on the concomitant MTX regimen. Additionally, we evaluated the PsA-specific composite measures such as MDA and DAPSA LDA (score ≤ 14) responses, which reflect therapeutic thresholds of LDA that patients achieve. We found that similar proportions of patients who achieved both MDA and DAPSA LDA responses showed improvement in both IXE regimens regardless of MTX. Overall, the addition of MTX does not improve the efficacy of IXE up to 1 year of treatment.

While this post hoc analysis was not powered to detect statistical differences between IXE treatment arms, there was no apparent increased benefit with IXE Q2W relative to IXE Q4W in arthritis-related measures. Dose-ranging studies in arthritis clinical trials have previously demonstrated that increased dose frequency does not necessitate improved therapeutic benefits [20, 28].

The extent of improvements observed in this post hoc analysis was similar to the observations from SPIRIT-P1 and SPIRIT-P2 trials evaluating the efficacy of IXE whether used alone or with any concomitant background cDMARDs (including MTX) up to 24 weeks of treatment [21, 22]. Methotrexate is often used off-label for the treatment of PsA in the clinical setting [29]. Despite its wide use, there is very limited evidence that supports efficacy of MTX in patients with PsA [30, 31]. The findings from our study are supported from randomized controlled trials with biologics, which demonstrate that clinically meaningful efficacy is achieved with or without background cDMARD [10, 11, 15, 32–34]. Most of these studies allowed the patients to continue background cDMARDs (including MTX). However, none of these trials were designed to directly compare the efficacy of biologics alone with concomitant cDMARDs, and the evidence from all these studies is mixed. In an open-label study comparing MTX monotherapy versus MTX along with infliximab in patients with PsA, the combination therapy demonstrated a significantly greater response for ACR20 and PASI 75 at week 16 but not for other parameters [32]. In the ADEPT trial, concomitant MTX with adalimumab showed little difference in responses up to week 12 irrespective of background MTX treatment, but the ACR20/50 responses were numerically better at week 48 in patients receiving adalimumab and concomitant MTX compared to adalimumab monotherapy [35]. The SEAM-PsA trial was specifically designed to compare the benefit of etanercept monotherapy with concomitant MTX. This study found that there was no additional benefit of added MTX to etanercept compared to etanercept monotherapy as measured by primary end points such as ACR and MDA response rates at week 24 [15]. Findings from other studies with concomitant MTX and secukinumab, another IL-17A antagonist, also showed no additional benefit in terms of efficacy [36, 37].

In a DANBIO study evaluating treatment response among Danish patients with PsA, improvement in ACR20 was seen but not in ACR50/70 with the combination treatment compared to the monotherapy group [14]. Furthermore, in the NOR-DMARD study evaluating the effect of concomitant MTX use in patients with PsA, no significant improvements in the ACR20/50/70 responses were seen with and without concomitant MTX use [10]. The studies from DANBIO and NOR-DMARD registries have shown higher long-term drug survival when using MTX background therapy [10, 14]. Results from the FUTURE 2 study found that the improvements were similar in both secukinumab with concomitant MTX and without MTX subgroups through week 104 [38]. In general, no additional benefit in efficacy or sustainability of response was seen with the addition of MTX to IXE, thus indicating that concomitant MTX use with IL-17 inhibitors might not have any additional benefit on clinical efficacy.

We have shown that the safety profile of IXE Q4W was similar between the monotherapy and concomitant MTX groups. A higher proportion of patients in IXE Q2W monotherapy experienced TEAEs compared to the concomitant MTX group. These findings are consistent with the previous reports for treatment with IXE in both PsA and psoriasis [39, 40]. Despite inadequate data to support the tolerability of MTX, it is often used for the management of PsA [6]. In patients with PsA, MTX has shown several safety concerns mainly being liver toxicity, which has been known to be exacerbated in patients with obesity, metabolic syndrome, and excess alcohol intake [41]. However, we did not observe differences in liver toxicity or hepatic abnormalities between patients with or without methotrexate in this study analysis.

The efficacy of biologics can be potentially affected by development of anti-drug antibody, and concomitant MTX administration decreases this development of anti-drug antibody for some biologics [42]. Previous findings showed that there was no clear distinction in treatment-emergent anti-drug antibodies between IXE-treated patients with or without concomitant MTX [43]. This parameter was not evaluated in our study.

The strength of the present analysis is that we examined patients who received MTX at baseline as well as those who were on a stable dose of MTX up to week 52. One of the limitations of this analysis is that it was conducted post hoc. Some patient’s baseline characteristics across treatment subgroups may have been imbalanced. This post hoc analysis could have also created unequal MTX dosing and administration route within treatment groups. Additionally, patients received MTX 12 weeks prior to randomization to IXE. There was no clear pattern whether more patients were tapering or had an increased uptake of MTX after week 24. The number of patients undergoing dose tapering/increase was small for a robust analysis, and their tapering/increase schedules varied. Hence, these results did not truly address the utility of MTX background therapy. Responses in MTX-naive patients may differ from those observed in this subset analysis. Radiographic progression of structural joint damage in patients with active PsA was assessed at weeks 24 in the SPIRIT-P1 trial and showed change from baseline in van der Heijde modified total Sharp score (mTSS) was significantly lower in patients treated with IXE Q4W or IXE Q2W with concomitant cDMARD or MTX use compared with their respective placebo-treated groups [21]. However, no structural data were collected in the SPIRIT-P2 trial.

## Conclusions

In this post hoc analysis, IXE showed improvements in efficacy with or without concomitant MTX therapy in patients with PsA up to 52 weeks of treatment. The safety profile is consistent with previous reports in patients with PsA and psoriasis [39, 40]. The findings of this study increase awareness of current treatment options and inform evidence-based treatment decisions when considering concomitant MTX use when prescribing IXE for patients with PsA. Further trial evaluating the efficacy and safety of IXE versus MTX as monotherapies and versus combination therapy in subjects with active psoriatic arthritis (PsA) could provide additional insights for clinical practice.

## Supplementary Information

**Additional file 1: Figure S1.** Disposition in the ITT population for patients randomized to (A) IXE Q4W and (B) IXE Q2W. Abbreviations: csDMARD=conventional synthetic disease-modifying antirheumatic drug; IXE Q2W=80 mg ixekizumab every 2 weeks; IXE Q4W=80 mg ixekizumab every 4 weeks; ITT=intent-to-treat; MTX=methotrexate; N=number of patients in the ITT population; Ns= number of patients in each category. **Figure S2.** Proportion of patients achieving ACR responses at Week 52 who were randomized to IXE and had concomitant MTX use at baseline. Abbreviations: ACR20/50/70=American College of Rheumatology criteria 20%/50%/70% improvement; cDMARD=conventional disease-modifying antirheumatic drug; IXE=ixekizumab; IXE Q2W=80 mg ixekizumab every 2 weeks; IXE Q4W=80 mg ixekizumab every 4 weeks; MTX=methotrexate. **Table S1.** Tender and swollen joint counts for patients receiving IXE with or without concomitant MTX treatment at week 52. Data are mean (standard deviation). Baseline is defined as the last non-missing value on or prior to the date of first study drug injection at Week 0 (Visit 2). ^a^Patients with stable dose of MTX from Weeks 0 to 52 only**.** Abbreviations: cDMARD=conventional disease-modifying antirheumatic drug; IXE Q2W=80 mg ixekizumab every 2 weeks; IXE Q4W=80 mg ixekizumab every 4 weeks; MTX=methotrexate; SJC=swollen joint count; TJC=tender joint count.

## Abbreviations

ACR: American College of Rheumatology
ACR20/50/70: American College of Rheumatology 20%/50%/70% response rate
AE: Adverse event
cDMARD: Convention disease-modifying antirheumatic drug
DANBIO: Danish Biologics
DAPSA: Disease activity in psoriatic arthritis
DMARD: Disease-modifying antirheumatic drug
EULAR: European League Against Rheumatism
IL: Interleukin
IXE: Ixekizumab
IXE Q2W: IXE 80 mg every 2 weeks
IXE Q4W: IXE 80 mg every 4 weeks
LDA: Low-disease activity
MIPA: Methotrexate in psoriatic arthritis
MTX: Methotrexate
MDA: Minimal disease activity
NOR-DMARD: Norwegian disease-modifying antirheumatic drug
PatGA: Patient’s Global Assessment of Disease Activity
PASI: Psoriasis Area and Severity Index
PsA: Psoriatic arthritis
SAE: Serious adverse event
SJC: Swollen joint count
TEAE: Treatment-emergent adverse even
TJC: Tender joint count
TNFi: Tumor necrosis factor inhibitor
VAS: Visual analog scale
