# Supplementary material for: Ixekizumab, with or without concomitant methotrexate, improves signs and symptoms of PsA: week 52 results from Spirit-P1 and Spirit-P2 studies
Source: Arthritis Res Ther. 2021 Jan 27;23:41. doi: 10.1186/s13075-020-02388-5 (PMC7839215; doi:10.1186/s13075-020-02388-5)
Supplement: Supplementary file 1 — Additional file 1: Figure S1. Disposition in the ITT population for patients randomized to (A) IXE Q4W and (B) IXE Q2W. Abbreviations: csDMARD=conventional synthetic disease-modifying antirheumatic drug; IXE Q2W=80 mg ixekizumab every 2 weeks; IXE Q4W=80 mg ixekizumab every 4 weeks; ITT=intent-to-treat; MTX=methotrexate; N=number of patients in the ITT population; Ns= number of patients in each category. Figure S2. Proportion of patients achieving ACR responses at Week 52 who were randomized to IXE and had concomitant MTX use at baseline. Abbreviations: ACR20/50/70=American College of Rheumatology criteria 20%/50%/70% improvement; cDMARD=conventional disease-modifying antirheumatic drug; IXE=ixekizumab; IXE Q2W=80 mg ixekizumab every 2 weeks; IXE Q4W=80 mg ixekizumab every 4 weeks; MTX=methotrexate. Table S1. Tender and swollen joint counts for patients receiving IXE with or without concomitant MTX treatment at week 52. Data are mean (standard deviation). Baseline is defined as the last non-missing value on or prior to the date of first study drug injection at Week 0 (Visit 2). aPatients with stable dose of MTX from Weeks 0 to 52 only. Abbreviations: cDMARD=conventional disease-modifying antirheumatic drug; IXE Q2W=80 mg ixekizumab every 2 weeks; IXE Q4W=80 mg ixekizumab every 4 weeks; MTX=methotrexate; SJC=swollen joint count; TJC=tender joint count. [file 13075_2020_2388_MOESM1_ESM.docx]

# ADDITIONAL FILE 1

**Figure S1**. **Disposition in the ITT population for patients randomized to (A) IXE Q4W and (B) IXE Q2W.**

Abbreviations: csDMARD=conventional synthetic disease‑modifying antirheumatic drug; IXE Q2W=80 mg ixekizumab every 2 weeks; IXE Q4W=80 mg ixekizumab every 4 weeks; ITT=intent-to-treat; MTX=methotrexate; N=number of patients in the ITT population; Ns= number of patients in each category.

**Figure S2**. **Proportion of patients achieving ACR responses at Week 52 who were randomized to IXE and had concomitant MTX use at baseline.**

Abbreviations: ACR20/50/70=American College of Rheumatology criteria 20%/50%/70% improvement; cDMARD=conventional disease‑modifying antirheumatic drug; IXE=ixekizumab; IXE Q2W=80 mg ixekizumab every 2 weeks; IXE Q4W=80 mg ixekizumab every 4 weeks; MTX=methotrexate.

**Table S1. Tender and swollen joint counts for patients receiving IXE with or without concomitant MTX treatment at week 52**

|  | **SPIRIT-P1 and SPIRIT-P2** | | | |
| --- | --- | --- | --- | --- |
|  | **IXE Q4W (N=229)** | | **IXE Q2W (N=226)** | |
|  | **No MTX/ cDMARDs** **(N=95)** | **MTX^a^**  **(N=85)** | **No MTX/ cDMARDs** **(N=82)** | **MTX^a^**  **(N=98)** |
| TJC, change from baseline | -12.6 (15.0) | -14.9 (13.8) | -17.8 (16.8) | -11.2 (11.7) |
| SJC, change from baseline | -7.5 (9.4) | -9.3 (10.3) | -10.5 (10.2) | -8.1 (7.9) |

Data are mean (standard deviation).

Baseline is defined as the last non-missing value on or prior to the date of first study drug injection at Week 0 (Visit 2).

^a^Patients with stable dose of MTX from Weeks 0 to 52 only

Abbreviations: cDMARD=conventional disease-modifying antirheumatic drug; IXE Q2W=80 mg ixekizumab every 2 weeks; IXE Q4W=80 mg ixekizumab every 4 weeks; MTX=methotrexate; SJC=swollen joint count; TJC=tender joint count.
